# The association between maternal and paternal parenting styles and adolescents’ overall mental problem: a longitudinal study

**DOI:** 10.1186/s40359-026-04525-2

**Published:** 2026-05-19

**Authors:** Daosen Wang, Ranran Gong, Chuang Liu, Kaize Yang, Xintong Su, Shihua Chen, Ruyi Ding, Yuhan Xing, Jiaxuan Fu, Xinyun Lin, Jingxian Zhao, Jie Tang, Qifei Chen, Xiuhong Li

**Affiliations:** 1 Department of Maternal, Child and Aging Health, School of Public Health (Shenzhen), Sun Yat-sen University, No.66, Gongchang Road, Guangming District, Guangdong, 518107 China; 2 Shenzhen Guangming District Maternal and Child Health Hospital, Guangdong, 518107 China; 3 Research Center of Children and Adolescent Psychological and Behavioral Development, Department of Maternal and Child Health, School of Public Health, Sun Yat-sen University, Guangzhou, 510080 China; 4 Department of Psychology, Sun Yat-Sen University, Guangzhou, 510080 China

**Keywords:** Parenting style, Adolescents, Epidemiology studies, Mental health

## Abstract

**Background:**

Adolescent mental problems are complex and increasingly prevalent. Parenting style is a key modifiable factor, yet differences between maternal and paternal influences remain underexplored.

**Methods:**

A cohort of 8,842 Chinese adolescents underwent dual-wave assessments (Sep 2022 to Sep 2023). Parenting styles were assessed using short Chinese version of the Egna Minnen av Barndoms Uppfostra scale. Mental problems were assessed using Mental Health Inventory of Middle School Students, Generalized Anxiety Disorder-7, and Patient Health Questionnaire-9. Mixed-effect models explored associations between parenting styles and adolescent’ mental problems. Latent transition analysis modeled mental problem profile dynamics.

**Results:**

Both paternal and maternal rejection and overprotection were associated with increased risks of multiple mental problems, whereas emotional warmth was protective. Latent profile analysis across ten domains (academic stress, maladjustment, hostility, psychological imbalance, interpersonal sensitivity, emotional instability, compulsiveness, paranoia, depression and anxiety) identified three profiles: low (L), moderate (M), and severe (S). Notably, paternal emotional warmth significantly improved overall mental problem trajectories (e.g. M→L: odds ratio [OR], 1.62; 95%CI, 1.19–2.21), whereas maternal rejection and over-protection predicted deterioration (e.g. L→M: rejection; OR, 2.52; 95% CI, 1.83–3.49 vs. over-protection; OR, 1.81; 95%CI, 1.29–2.54), with maternal rejection exerting the stronger adverse association.

**Conclusions:**

Maternal and paternal parenting styles exerted distinct influences on adolescent mental problems. Father’s emotional warmth was associated with more favorable overall mental problem trajectories, while maternal rejection and over-protection predicted poorer outcomes, particularly the rejection. These findings suggest that interventions should differentiate between maternal and paternal roles when addressing adolescent mental problems.

**Supplementary Information:**

The online version contains supplementary material available at 10.1186/s40359-026-04525-2.

## Introduction

Mental problems are highly prevalent during adolescence, with 25–50% of youths experiencing at least one disorder, among these, about 40% facing multiple problems simultaneously or sequentially [1–4]. These conditions contribute to 15–30% of disability-adjusted life-years (DALYs) lost in the first three decades of life, imposing considerable personal, social, and economic burdens by disrupting education and future employment [5, 6]. Consequently, research has increasingly focused on the etiological factors underlying adolescent mental problems.

Beyond genetic predispositions, modifiable environmental factors, particularly parenting styles, play a critical role [7, 8]. Parenting styles-defined as consistent attitudes, goals, and practices-affect adolescents’ emotional regulation and neural development [9, 10]. Positive parenting styles, such as emotional warmth, have been identified as protective factors against a range of adolescent mental problems, including both internalizing difficulties (e.g., anxiety and depression) and externalizing behaviors [11]. In contrast, negative parenting styles, including rejection and overprotection, are associated with increased risks for internalizing symptoms, externalizing behaviors, and long-term dysfunction [12, 13].

However, most studies have focused on mothers or aggregated parental data, overlooking distinct maternal and paternal roles [14]. Evidence suggests that mothers tend to be more responsive caregivers, while fathers often promote risk-taking and resilience, leading to potentially different effects of similar parenting styles on adolescent mental problems [12, 15, 16]. For example, one review found that maternal over-protection may play a more important role in predicting adolescent anxiety than paternal over-protection [17].This may be because mothers’ caregiving roles often require them to invest more in primary care and lead to more overprotective behavior [18]. In contrast, fathers are thought to encourage their children’s risk-taking behaviors and reduce their anxiety levels [12].

Furthermore, adolescent mental problems are complex and various, and exhibit substantial heterogeneity, characterized by diverse symptom patterns, high comorbidity (such as depression and anxiety) [19–22]. Adolescents with multiple psychological problems often experience poorer outcomes and significant deficits across various cognitive and behavioral domains [23]. However, the majority of existing studies have examined associations between parenting styles and specific, isolated mental health outcomes (e.g., anxiety or depression alone) [9, 24]. Measuring the severity of a single mental problem in isolation is often insufficient to capture the true clinical picture; for instance, an adolescent might present with “mild” symptoms across multiple distinct domains, but the cumulative effect of these overlapping issues may result in a moderate-to-severe overall mental health burden [25]. To adequately address this heterogeneity in symptom presentation, person-centered approaches such as Latent Profile Analysis (LPA) have proven particularly valuable, as they identify subgroups of adolescents who share similar profiles of co-occurring mental problems [26, 27]. According to the person-oriented theoretical framework [28], the optimal number of latent profiles balances statistical fit indices with substantive criteria: yielding a limited, parsimonious set of meaningful, homogeneous subgroups reflecting expected patterns of symptom comorbidity, while remaining interpretable, distinct across time, and linked to external covariates such as parenting styles [29]. Based upon LPA, Latent Transition Analysis (LTA) extends this framework longitudinally by modeling how individuals transition between these latent classes over time [30]. Understanding how class membership changes over time is of paramount importance because adolescent mental health is highly dynamic rather than static; tracking these longitudinal shifts allows researchers to identify critical windows of risk escalation or natural symptom remission [26, 27]. Furthermore, it is crucial to understand how parenting relates to these changes, as the family environment remains a primary, malleable context during this sensitive developmental period [30, 31]. Specific parenting behaviors can act as critical catalysts for these transitions: positive practices like emotional warmth may foster resilience and facilitate recovery (i.e., transitioning into lower-risk profiles), whereas negative styles such as rejection and overprotection may exacerbate psychological vulnerabilities (i.e., transitioning into higher-risk profiles) [13, 32]. This dynamic perspective is essential because it reflects the real-world complexity of adolescent mental problems and allows researchers to examine whether and how parenting styles influence the probabilities of transitioning into or out of high-risk symptom profiles [33]. Investigating these parenting-related mental problem transition patterns can better identify how parenting styles contribute to overall mental problem trajectories, reveal patterns of co-development across problems, and inform targeted, multifaceted interventions that promote resilience across a range of symptoms rather than isolated conditions [34]. This approach is particularly crucial for guiding global prevention and treatment strategies that account for the cumulative and interactive effects of mental health challenges.

To address these gaps, our study investigates the association between father’s and mother’s parenting styles and adolescent comprehensive mental problem transitions. Specifically, we aim to: (1) Explore the longitudinal associations between parenting styles and adolescents’ mental problems using a variable-centered approach. We hypothesize that both fathers’ and mothers’ positive parenting styles are associated with a reduction in adolescent mental problems, while negative parenting styles are associated with an increase in these problems. (2) Identify adolescents’ overall mental problem trajectories based on ten specific symptoms using a person-centered LPA method. We hypothesize that there will be multiple trajectories of adolescent mental problems and that these trajectories will exhibit a trend of gradual worsening over time. (3) Investigate the separate associations between fathers’ and mothers’ parenting styles and the identified mental problem trajectories and transitions based on a person-centered LTA method. We hypothesize that mothers’ and fathers’ parenting styles will have significant associations with adolescents’ membership in and transitions between these mental problem trajectories. In addition, to provide a more comprehensive understanding using complementary person-centered approaches, supplemental analyses examined (a) cross-sectional associations between parenting styles and mental problem profiles, (b) associations between one-year changes in parenting styles and changes in mental problem profiles, (c) associations between baseline parenting style scores and one year follow-up mental problem profiles. We hope our findings will offer valuable insights into this understudied area and inform interventions for adolescent mental health globally based on parenting styles.

## Method

### Study population

Data were derived from a longitudinal survey conducted in Shenzhen, Guangdong Province, China. Students from 27 middle schools and 3 high schools were recruited using cluster sampling. Two waves of data collection were conducted in September 2022 (grades 7, 8, 10, and 11) and September 2023.

A total of 8964 students completed both waves of the survey. After excluding incomplete responses, the final sample consisted of 8842 adolescents. The study was approved by the Human Studies Committee at Sun Yat-sen University. Informed consent was obtained from both parents of the participating adolescents, the participating school psychologists and the participating adolescents. Students received age-appropriate information about the study purpose, procedures, voluntary nature of participation, and their right to decline or withdraw at any time without consequence.

### Covariates

Adolescents completed a demographic questionnaire, which included age, sex, preterm birth, parental education levels, only-child status, and single-parent family.

### Assessment of adolescent mental health

The Mental Health Inventory of Middle School Students (MMHI) was used to assess eight specific dimensions of adolescent mental health. Based on previous research [35], these subscales are defined as follows: academic stress (perceived pressure from academic demands, exams, and school performance), maladjustment (difficulties adapting to school life, teachers, peers, and the broader environment), hostility (anger, irritability, and aggressive impulses toward others), psychological imbalance (a sense of emotional or cognitive disequilibrium, feelings of unfair treatment by authority figures, and resentment toward peers who outperform oneself), interpersonal sensitivity (heightened sensitivity to others’ opinions, criticism, or social rejection), emotional instability (frequent mood fluctuations and challenges in emotional regulation), compulsiveness (obsessive thoughts and repetitive, ritualistic behaviors), and paranoia (suspiciousness and paranoid ideation regarding others’ intentions). Each subscale consists of six items, with the mean score used to classify mental problems as follows: no problems (mean < 2:); mild problems (mean 2-2.99); moderate problems (mean 3-3.99); severe problems (mean 4-4.99); serious problems (mean 5-5.99). The MMHI has been validated for use in Chinese adolescents, demonstrating high reliability (retest reliability = 0.72–0.91) and structural validity (0.47–0.76) [36]. In the present sample, the MMHI subscales demonstrated acceptable to excellent internal consistency, with Cronbach’s α at Wave 1 (2022) and Wave 2 (2023) as follows: academic stress (0.88/0.89), maladjustment (0.80/0.82), hostility (0.88/0.89), psychological imbalance (0.79/0.81), interpersonal sensitivity (0.81/0.83), emotional instability (0.84/0.87), compulsiveness (0.75/0.78), and paranoia (0.85/0.87).

Anxiety symptoms were assessed using the Generalized Anxiety Disorder 7-item Scale (GAD-7) which is commonly used in the adolescents [37]. It includes seven items measuring the severity of anxiety symptoms. Total scores range from 0 to 21, with severity classified as: no anxiety (0–4), mild anxiety (5–9), moderate anxiety (10–14), and severe anxiety (15–21). The GAD-7 has demonstrated excellent internal consistency (Cronbach’s α = 0.93–0.95) and construct validity (factor loadings > 0.6) in Chinese adolescents [38]. In the current sample, the GAD-7 demonstrated excellent internal consistency (Cronbach’s α = 0.93 in 2022 and 0.94 in 2023).

Depression symptoms were measured using the Patient Health Questionnaire Depression Scale (PHQ-9) [39]. Each item is scored on a 4-point scale, with total scores ranging from 0 to 27. Severity is classified as: no depression (0–4); mild depression (5–9), moderate depression (10–14), moderately severe depression (15–19); severe depression (≥ 20). The PHQ-9 has demonstrated strong reliability (Cronbach’s α = 0.86) and test-retest validity in Chinese adolescents (*r* = 0.86) [40]. In the current sample, the PHQ-9 demonstrated excellent internal consistency (Cronbach’s α = 0.93 in 2022 and 0.94 in 2023).

### Assessment of parenting style

Parenting styles were assessed via adolescent self-report using the short Chinese version of the Egna Minnen Beträffande Uppfostran (s-EMBU-C), which measures three dimensions of parenting: rejection (e.g. hostility, punishment), emotional warmth (e.g. special attention, praise), and overprotection (e.g. intrusiveness). Each dimension was assessed separately for fathers and mothers with items scored on a 4-point scale from “never” (1) to “always” (4). The s-EMBU-C has demonstrated acceptable reliability (Cronbach’s α = 0.74–0.84) and criterion validity (0.82–0.93) in Chinese adolescents [41]. In the present sample, the s-EMBU-C subscales demonstrated acceptable to excellent internal consistency, with Cronbach’s α at 2022 and 2023 as follows: fathers’ rejection (0.85/0.85), fathers’ emotional warmth (0.89/0.89), fathers’ over-protection (0.71/0.70), mothers’ rejection (0.84/0.85), mothers’ emotional warmth (0.88/0.87), mothers’ over-protection (0.73/0.72).

### Statistical analysis

#### Variable-centered analysis

To address our first aim, we examined the associations between parenting styles and adolescents’ mental problems.

To avoid severe multicollinearity issues driven by the high intercorrelations between maternal and paternal parenting styles (e.g., *r* = 0.86 for emotional warmth, *r* = 0.84 for over-protection, and *r* = 0.76 for rejection), six separate multilevel random effects models were used to examine the longitudinal associations between specific parenting styles and adolescents’ mental problems across the two waves (eTable3). In each model, year was used as a random slope and schools as a random intercept. Covariates included adolescents’ age, sex, preterm birth status, parental education levels, only-child status, and single-parent family status.

#### Person-centered analyses

To address our second aim, LPA was conducted separately on baseline and one-year follow-up ratings from the MMHI subscales, GAD-7, and PHQ-9. Models with 2–6 profiles were tested, fit indices are shown in Table 3. Estimation was halted after the 6-profile model because the size of the smallest extracted classes had dropped to unacceptably low levels (2.99% at baseline and 2.08% at follow-up), falling well below the recommended 5% threshold required to ensure computational stability in the subsequent Latent Transition Analysis. Estimating a 7-profile model would have only further fragmented the sample into theoretically meaningless subgroups. The 3-profile solution was selected as optimal at both waves because: (a) it yielded the largest reductions in AIC, BIC, and SABIC relative to the 2-profile model (e.g., baseline BIC drop of 6,472 points); (b) it produced the highest entropy (0.93 baseline, 0.94 follow-up), indicating superior classification quality; (c) the smallest class remained adequately sized (12.25% baseline, 14.38% follow-up; >1,000 cases each), whereas 5 and 6 profile solutions produced classes < 5% that risk instability in LTA; (d) the Lo-Mendell-Rubin test remained significant [42]. Selection of the optimal model was further informed by prior theory, interpretability of the profiles, and their distinctiveness across time points [43, 44].

To further explore changes in the trajectories of mental problems, LTA, based on the latent Markov model, was employed to explore transitions across mental problem profiles over time [45]. To ensure that the latent profiles maintained the same substantive meaning over time, measurement invariance across the baseline and follow-up time points was strictly tested. This was achieved by comparing an unconstrained latent transition model with a constrained model (where item response parameters were equated across waves) using the Satorra-Bentler scaled χ^2^ difference test [46]. Detailed analytical procedures and the R code demonstrating the implementation of this invariance testing via the MplusAutomation package are provided in Supplementary Appendix.

### Baseline parenting styles associated with transitions in mental problem profiles

To address our third aim, parenting style scores were modeled as continuous predictors of profile transition probabilities via logistic regression. Additional covariates, including adolescents’ age, sex, school, preterm, parental education levels, only-child status, and single-parent family, were included in the model.

### Cross-sectional associations between parenting styles and mental problem profiles

Multivariate logistic regression was used to examine the association between parenting styles (continuous variables) and profile membership (categorical variable) at both time points, with the low mental problem profile as the reference group. Covariates included adolescents’ age, sex, school, preterm birth status, parental education levels, only-child status, and single-parent family status.

### Associations between baseline parenting styles and one year follow up mental problem profiles

Multivariate logistic regression was used to examine the association between baseline parenting styles (continuous variables) and one year follow up profile membership (categorical variable), with the low mental problem profile as the reference group. Covariates included adolescents’ age, sex, school, preterm birth status, parental education levels, only-child status, and single-parent family status.

### Associations between change in mental problems and change in parenting styles

For each separate mental problem transition pattern (*n* = 9) and parenting style scores (*n* = 6), one-sample t tests were run to determine if the change in parenting style scores (follow-up subtracted by baseline) was significantly different from zero.

Statistical significance was set at *P* < 0.05, with all *P*-values two-tailed. LPA, LTA, and logistic regression analyses were conducted using R version 4.4.1.

## Results

### Demographic characteristics of the sample

As shown in Table 1, the study included 8842 participants with a mean age of 13.4 years. Among them, 4747 (53.7%) were boys, and 385 (4.4%) were premature infants. Regarding parental education, 6018 fathers (68.1%) and 6116 mothers (69.2%) had educational attainment below high school. Additionally, 1,486 (16.8%) participants were only children, and 369 (4.2%) came from single-parent families at baseline. Details on adolescents’ mental problems and parenting style scores are provided in eTable1 and eTable2. At both baseline and one-year follow-up, academic stress was the most prevalent adolescent mental problem, affecting 47.16% and 51.88% of adolescents at mild or higher levels, respectively. In contrast, psychological imbalance had the lowest prevalence, with 18.25% and 21.12% of adolescents reporting mild or higher levels, respectively. Among parenting styles, emotional warmth exhibited the highest scores for both fathers and mothers at both time points.

**Table 1:** Description of the analytic sample^a^

Variable	Baseline (*n* = 8842)	One year follow-up (*n* = 8842)	*P*-value
Age, mean (SD), year	13.4 (1.3)	14.4 (1.3)	< 0.01
Sex, No. (%)			-
Boy	4747 (53.7%)	4747 (53.7%)	
Girl	4095 (46.3%)	4095 (46.3%)	
Preterm birth, No. (%)			-
Yes	385 (4.4%)	385 (4.4%)	
No	8457 (95.6%)	8457 (95.6%)	
Father’s education level, No. (%)			-
≤high school	6018 (68.1%)	6018 (68.1%)	
> high school	2824 (31.9%)	2824 (31.9%)	
Mother’s education level, No. (%)			-
≤high school	6116 (69.2%)	6116 (69.2%)	
> high school	2726 (30.8%)	2726 (30.8%)	
Only child, No. (%)			0.75
Yes	1486 (16.8%)	1470 (16.6%)	
No	7356 (83.2%)	7372 (83.4%)	
Single-parent families, No. (%)			0.08
Yes	369 (4.2%)	417 (4.7%)	
No	8473 (95.8%)	8425 (95.3%)	
Father’s rejection score	1.53 (0.58)	1.49 (0.55)	< 0.01
Father’s emotional warmth score	2.48 (0.76)	2.48 (0.76)	0.85
Father’s over-protection score	1.98 (0.52)	1.99 (0.51)	0.56
Mother’s rejection score	1.54 (0.56)	1.49 (0.52)	< 0.01
Mother’s emotional warmth score	2.59 (0.75)	2.60 (0.74)	0.51
Mother’s over-protection score	2.07 (0.54)	2.07 (0.53)	0.57

*Abbreviation*: Standard deviation

### Variable-centered analysis: associations between parenting styles and adolescents’ mental problems

As shown in Table 2, both father’s and mother’s rejection and over-protection were associated with increased risks across all dimensions of adolescents’ mental problems. Additionally, the associations between maternal rejection and adolescent mental health problems were consistently stronger than those for maternal over-protection across all ten mental problem indicators. For fathers, a similar pattern emerged for the majority of outcomes (9 out of 10 indicators); however, paternal over-protection was more strongly associated with compulsiveness than was paternal rejection. Furthermore, the associations between maternal rejection and all ten mental problem outcomes were consistently stronger than those for paternal rejection. While maternal over-protection showed stronger associations with seven of the mental health outcomes compared to paternal over-protection, paternal over-protection showed stronger associations with paranoia, anxiety, and psychological imbalance.

**Table 2:** Association between parenting style scores and adolescents’ mental problem^ab^

OR [95%CI]	Mental problems in different dimensions									
	Obsessive-Compulsive tendencies	Paranoid Ideation	Hostility	Interpersonal Sensitivity	Academic Stress	Maladaptation	Anxiety	Depression	Emotional Disturbance	Psychological Imbalance
Father’s parenting style										
Rejection	3.04 (2.85, 3.23)	4.02 (3.76, 4.29)	3.76 (3.52, 4.01)	3.86 (3.62, 4.12)	3.30 (3.08, 3.53)	3.54 (3.32, 3.77)	3.73 (3.49, 3.99)	4.46 (4.15, 4.79)	4.35 (4.05, 4.66)	3.75 (3.51, 4.01)
Emotional warmth	0.72 (0.69, 0.75)	0.56 (0.53, 0.59)	0.56 (0.53, 0.59)	0.58 (0.55, 0.60)	0.59 (0.57, 0.62)	0.55 (0.52, 0.57)	0.59 (0.56, 0.61)	0.55 (0.53, 0.57)	0.55 (0.53, 0.57)	0.60 (0.56, 0.63)
Over-protection	3.14 (2.94, 3.36)	3.45 (3.22, 3.70)	3.34 (3.12, 3.58)	3.44 (3.21, 3.68)	2.88 (2.70, 3.08)	2.90 (2.71, 3.10)	3.50 (3.26, 3.74)	3.86 (3.59, 4.14)	3.51 (3.28, 3.77)	3.50 (3.25, 3.77)
Mother’s parenting style										
Rejection	3.26 (3.06, 3.48)	4.34 (4.05, 4.65)	4.21 (3.93, 4.51)	4.33 (4.05, 4.64)	3.74 (3.49, 4.01)	3.82 (3.57, 4.08)	4.11 (3.84, 4.41)	5.11 (4.74, 5.50)	4.93 (4.58, 5.30)	4.35 (4.05, 4.67)
Emotional warmth	0.75 (0.72, 0.79)	0.58 (0.55, 0.61)	0.57 (0.54, 0.60)	0.59 (0.57, 0.62)	0.61 (0.59, 0.64)	0.57 (0.54, 0.59)	0.59 (0.57, 0.62)	0.58 (0.55, 0.60)	0.57 (0.55, 0.59)	0.57 (0.54, 0.60)
Over-protection	3.21 (3.01, 3.43)	3.39 (3.17, 3.63)	3.35 (3.13, 3.58)	3.49 (3.27, 3.73)	2.97 (2.78, 3.17)	2.96 (2.78, 3.16)	3.42 (3.20, 3.65)	4.00 (3.74, 4.29)	3.63 (3.40, 3.88)	3.42 (3.18, 3.68)

^a^Model uses the survey year as the random slope and the school as the random intercept and was adjusted for child age, sex, preterm birth, parental education levels, only child, single-parent families

^b^All *P value* < 0.05

In terms of protective parenting styles, both paternal and maternal emotional warmth were significantly associated with a reduced risk of adolescents’ mental health problems. The magnitude of these protective effects was comparable between parents; however, a detailed examination of the estimates revealed that paternal emotional warmth showed stronger protective associations across 8 of the 10 mental problems (e.g., for compulsiveness, the protective estimate was OR = 0.72 for fathers versus OR = 0.75 for mothers).

### Person-centered analysis: adolescent mental problem profiles and transitions

The fit indices for the LPA models at baseline and one-year follow-up are shown in Table 3. The AIC, BIC, BIC-SA values consistently decreased as the number of latent classes increased, and the *P-values* for the LMRT remained below 0.01 at both time points, suggesting that a six-profile solution was optimal. However, the three-class model achieved the highest entropy at both time points. Considering model simplicity and interpretability, the three-class solution was selected, as higher entropy indicates better classification accuracy [44].

**Table 3:** Model fit statistics for latent profile analyses at baseline and one year follow-up

	AIC	BIC	BIC-SA	Size of smallest class	Entropy	*P*-value of LMRT
Baseline						
2-class	122,209	122,769	122,605	35.47%	0.90	0.01
3-class	114,454	115,297	115,050	12.25%	**0.93**	0.01
4-class	111,905	113,032	112,701	8.07%	0.85	0.01
5-class	111,169	112,580	112,166	6.46%	0.84	0.01
6-class	**110,375**	**112,069**	**111,572**	2.99%	0.77	0.01
One year follow-up						
2-class	127,880	128,440	128,276	39.63%	0.92	0.01
3-class	118,545	119,389	119,141	14.38%	**0.94**	0.01
4-class	115,919	117,046	116,716	10.73%	0.85	0.01
5-class	114,811	116,221	115,807	4.35%	0.78	0.01
6-class	**113,966**	**115,660**	**115,163**	2.08%	0.86	0.01

*Abbreviation*: *AIC* Akaike Information Criterion, *BIC* Bayesian Information Criterion, *BIC-SA* Bayesian Information Criterion - Sample-size adjusted, *LMRT* Lo-Mendell-Rubin Likelihood Ratio Test. Values in bold indicate the optimal model based on the criterion for that column

**Fig. 1:**
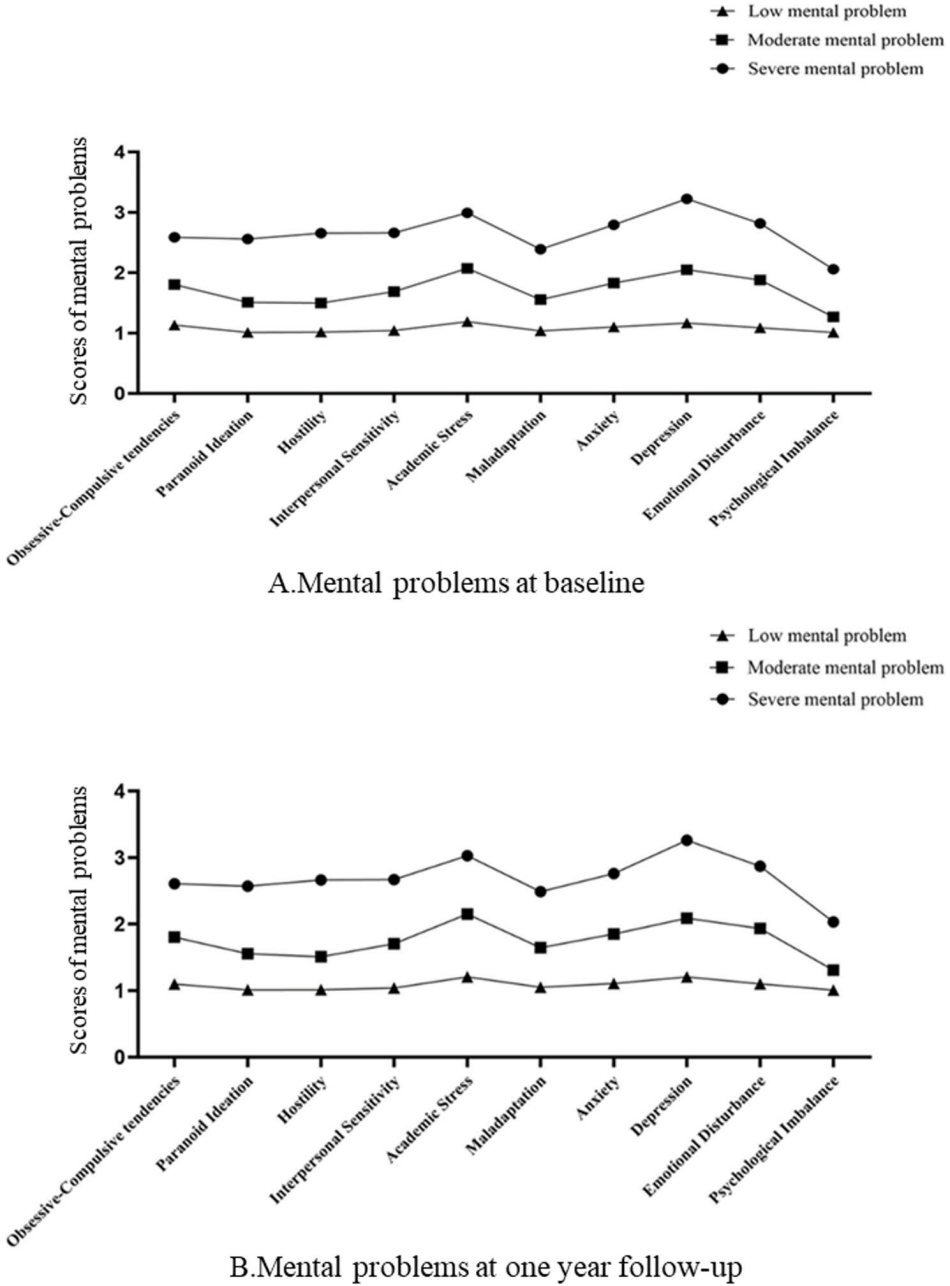
Adolescents’ mental problem profiles at baseline and one year follow-up^a^.^a^Mean scores on subscales of the Mental Health Inventory of Middle School Students (MMHI-60), the Generalized Anxiety Disorder 7-item Scale (GAD-7), and the Patient Health Questionnaire Depression Scale (PHQ-9) for each adolescents’ mental problem profile at baselineaa

Conditional item scores for the three-profile solution are presented in Figs. 1, illustrating distinct mental problem profiles among adolescents. The LPA results revealed consistent patterns across time. At both baseline and follow-up, class 1 was labeled as the “low mental problem group” (L) due to its low scores across all indicators. Conversely, class 3, the “severe mental problem group” (S) displayed the highest scores across all indicators, while class 2, the “moderate mental problem group” (M) fell between the two extremes. The Satorra-Bentler chi-square difference test confirmed the assumption of measurement invariance across time (eTable4), indicating that the profiles’ meaning and characteristics remained stable.

**Fig. 2:**
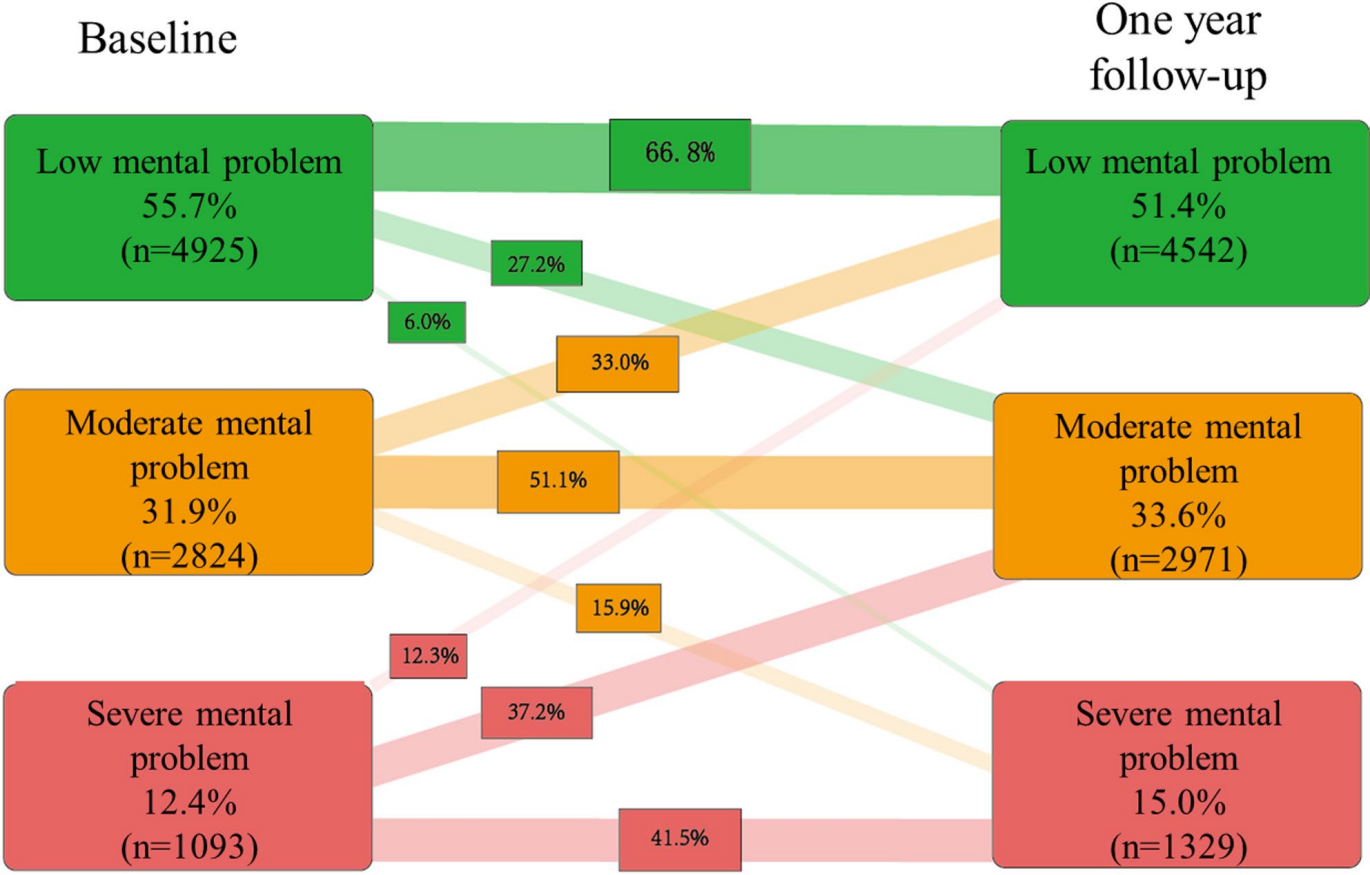
Transitions between adolescents’ mental problem profiles from baseline to one year follower-up^a^.^a^ Transitions between adolescents’ mental health profiles from baseline (left) to one year follow-up (right). Percentages and line thickness represent the proportions of each profile that remained in the same class over time or transitioned to a different profile

As illustrated in Fig. 2, the distribution of participants across profiles showed that, at baseline, 4925 participants (55.7%) were in the low mental problem group, 2824 (31.9%) in the moderate group, and 1093 (12.4%) in the severe group. At follow-up, the proportions were 4542 (51.4%), 2971 (33.6%), and 1329 (15.0%) in the low, moderate, and severe mental problem groups, respectively.

Transition probabilities, also shown in Fig. 2, reveal a nuanced pattern of both structural continuity and clinically meaningful mobility over the one-year period. While the highest probabilities were generally observed on the diagonal (indicating that a large proportion of adolescents remained in their baseline profiles), the off-diagonal transition rates were substantial. Specifically, transitions between adjacent profiles were relatively common: 37.2% of adolescents in the severe mental problem group transitioned to the moderate group, and 27.2% of those in the low group transitioned to the moderate group. In contrast, extreme shifts were far less frequent, with only 6.0% transitioning directly from the low to the severe group.

To quantify the overall deterioration of mental problem trajectories over the one-year period, we calculated the number and percentage of adolescents who transitioned from a lower-symptom class at baseline to a higher-symptom class at follow-up. Based on the transition probabilities, approximately 23.6% of the total sample (*n* = 2084) experienced a worsening of mental problem. Specifically, 1340 adolescents transitioned from the low to the moderate mental problem group (27.2% of the baseline low group), 296 transitioned from the low to the severe mental problem group (6.0% of the baseline low group), and 449 transitioned from the moderate to the severe mental problem group (15.9% of the baseline moderate group). In contrast, only 16.7% of the total sample (*n* = 1473) transitioned to a lower-symptom class, empirically supporting an overall trend of deterioration in adolescent mental health trajectories.

### Person-centered analysis: associations between parenting styles and adolescent mental problem profiles

As shown in Table 4, father’s emotional warmth was associated with improved mental problem profiles (M→L: OR, 1.62; 95%CI, 1.19–2.21) and prevention of mental problem deterioration (L→M: OR, 0.60; 95%CI, 0.47–0.76). Conversely, maternal rejection and over-protection increase the risk of worsening mental problem profiles (L→M: rejection; OR, 2.52; 95% CI, 1.83–3.49 vs. over-protection; OR, 1.81; 95%CI, 1.29–2.54) and decrease the chances of mental problem improvement (M→L: rejection; OR, 0.33; 95% CI, 0.21–0.53 vs. over-protection; OR, 0.52; 95%CI, 0.32–0.83), with maternal rejection being more harmful.

**Table 4:** Associations between parenting style scores and transition patterns of adolescents’ latent mental problem profile ^a b^

Parenting styles	Transition pattern [OR (95%CI)]		
	L→L	L→M	L→S
Father’s rejection	Ref.	1.57 (0.94, 2.61)	1.56 (0.85, 2.86)
Mother’s rejection	Ref.	**2.52 (1.83**,** 3.49)**	**3.70 (2.00**,** 6.84)**
Father’s over-protection	Ref.	1.18 (0.83, 1.67)	1.24 (0.62, 2.48)
Mother’s over-protection	Ref.	**1.81 (1.29**,** 2.54)**	1.16 (0.59, 2.28)
Father’s emotional warmth	Ref.	**0.60 (0.47**,** 0.76)**	0.86 (0.49, 1.52)
Mother’s emotional warmth	Ref.	1.11 (0.87, 1.43)	0.59 (0.33, 1.05)
	M→M	M→L	M→S
Father’s rejection	Ref.	0.75 (0.48, 1.15)	1.61 (0.92, 2.83)
Mother’s rejection	Ref.	**0.33 (0.21**,** 0.53)**	**1.50 (1.02**,** 2.20)**
Father’s over-protection	Ref.	1.10 (0.68, 1.77)	0.93 (0.60, 1.44)
Mother’s over-protection	Ref.	**0.52 (0.32**,** 0.83)**	1.51 (0.98, 2.32)
Father’s emotional warmth	Ref.	**1.62 (1.19**,** 2.21)**	1.05 (0.76, 1.47)
Mother’s emotional warmth	Ref.	0.73 (0.53, 1.01)	0.76 (0.54, 1.07)
	S→S	S→L	S→M
Father’s rejection	Ref.	0.49 (0.21, 1.13)	0.91 (0.57, 1.45)
Mother’s rejection	Ref.	**0.22 (0.09**,** 0.52)**	**0.49 (0.30**,** 0.80)**
Father’s over-protection	Ref.	1.75 (0.68, 4.46)	0.87 (0.51, 1.48)
Mother’s over-protection	Ref.	**0.24 (0.10**,** 0.58)**	0.66 (0.40, 1.09)
Father’s emotional warmth	Ref.	**2.42 (1.30**,** 4.53)**	1.06 (0.70, 1.59)
Mother’s emotional warmth	Ref.	0.36 (0.13, 1.01)	0.98 (0.65, 1.48)

*Abbreviation*: *CI* Confidence interval; *L* low mental problem profile, *M* Moderate mental problem profile, *S* Severe mental problem profile

^a^ Model was adjusted for school, child age, sex, preterm birth, parental education levels, only child, single-parent families

^b^ Bold indicates that the *P value* < 0.05

Similar results were also found in longitudinal analysis, cross-sectional analysis and one-sample t-tests. Higher rejection and over-protection scores were associated with greater odds of being classified into moderate or severe mental problem profiles, while higher emotional warmth scores were linked to lower odds of severe profiles (eTable5). Transitions to more severe profiles were associated with increases in rejection and over-protection and decreases in emotional warmth, while transitions to less severe profiles showed the opposite pattern (eTable6). Higher rejection and over-protection scores at baseline were associated with greater odds of being classified into moderate or severe mental problem profiles at follow up, while higher emotional warmth scores at baseline were linked to lower odds of severe profiles at follow up (eTable7).

## Discussion

This study notably described adolescents’ overall mental problem trajectories using an individual-centered approach and examined the differential associations of maternal versus paternal parenting styles within a large longitudinal sample. The important findings included: (1) based on our variable-centered analysis, both fathers and mothers’ rejection and over-protection were associated with increased adolescent mental problems across all dimensions, whereas emotional warmth was protective. Compared with mothers, fathers’ rejection and over-protective were less associated with adolescent mental problems, while paternal warmth exhibited stronger protective associations. Our variable-centric analysis results, both cross-sectional and longitudinal, support the above findings. (2) Integrating ten mental problems, we identified three profiles, namely low, moderate, and severe mental problem profile, highlighting the high comorbidity of adolescent mental problems. 3). Third, our person-centered analysis revealed that paternal emotional warmth predicted more favorable developmental trajectories, while maternal rejection and overprotection, particularly rejection, predicted transitions toward poorer outcomes. This study not only emphasizes the importance of maternal parenting styles, but also highlights the different roles and significance of paternal parenting styles on adolescent overall mental problems which have important public health significance.

Our variable-centered analysis results yielded results consistent with prior research. We found that negative parenting styles exacerbate adolescent mental problems, while emotional warmth reduces risk [47, 48]. The underlying mechanisms of these findings are still unclear, but some studies suggest that they may be related to adolescent emotion processing and regulation and related neural circuits [49]. For example, anxiety has been linked to imbalances in emotional processing capacity, such as hypervigilance to threat and difficulties in regulation [50]. Warm parenting has been associated with reduced activation in emotional salience regions when responding to negatively valenced stimuli, suggesting attenuated reactivity to emotional threats while harsh or rejecting parenting has been linked to reduced activation in regions responsible for emotion regulation [51, 52]. This may partly explain our findings.

We also found that compared with mothers, fathers’ rejection and over-protective were weakly associated with increased adolescent mental problems, while emotional warmth was strongly associated with decreased adolescent mental problems. Similar to our results, some studies also found mothers’ negative parenting style is more harmful to adolescents’ mental health than fathers’ [53]. This may be because mothers place a greater emphasis on protection, care, and maintaining close relationships than fathers. As a result, mothers’ heightened concern and overprotectiveness inhibit the development of coping skills and autonomy, leading to more mental problems [54]. What’s more, compared to fathers, mothers’ role is to provide nurturing and care for their children in Chinese culture [55]. When teenagers enter middle school and high school, they will be more interfered with and punished by their mothers rather than fathers, which will have a more serious negative impact on their mental development [56]. However, there is also studies that found no differences in the associations between maternal and paternal over-protection and mental problems in their offspring, which may be due to the fact that the analysis was restricted to children, while our study focused specifically on adolescents [57]. In terms of positive parenting styles, an intervention study found that fathers who take more active parenting actions are more likely to reduce children’s internalizing behaviors than mothers [58]. Its potential mechanism may be attributed to the differing parenting characteristics, where fathers are more inclined to adopt rejecting or excessive styles, while mothers tend to exhibit warmer behaviors [59]. As a result, adolescents may be more sensitive to paternal warmth and less responsive to maternal warmth [60].

Notably, our person-centered analysis identified three mental health profiles: low, moderate, and severe, indicating that the heterogeneity in our sample was driven primarily by varying levels of overall symptom severity rather than qualitatively diverse, symptom-specific patterns. This finding suggests that adolescent mental health problems in this population are highly comorbid; we did not observe subgroups characterized by a single dominant mental problem. Importantly, this quantitative accumulation underscores the limitation of variable-centered approaches: the severity of an isolated mental health issue might not adequately represent the true psychological burden when multiple conditions co-occur. For example, while individual symptoms may only reach a “mild” clinical threshold when evaluated separately, their combined presence can elevate the adolescent into a “moderate” or “severe” overall risk profile [25]. This aligns with prior research demonstrating that mental health challenges during adolescence rarely occur in isolation, and it is the cumulative severity of these co-occurring symptoms that often dictates the need for comprehensive interventions [61]. This may be due to delayed brain development during adolescent which may contributing to hyperconnectivity of prefrontal-related neural circuits and ultimately lead to various mental problems [62]. In addition, we also found that adolescents’ mental problem trajectories also deteriorated over time, which may be influenced by increasing age-related challenges such as academic pressure and evolving social relationships [63].

Regarding protective parenting styles, our cross-sectional analyses indicated that both maternal and paternal emotional warmth were significantly associated with more favorable adolescent mental health profiles, consistently reducing the likelihood of belonging to the moderate or severe symptom groups. However, when examining longitudinal transition patterns, an important distinction emerged: paternal emotional warmth was uniquely associated with positive changes in symptom profiles over time. While maternal warmth did not significantly predict longitudinal shifts, higher levels of paternal emotional warmth significantly increased the probability of adolescents transitioning from high-risk or moderate-risk profiles to the low-risk profile. This suggests that while both parents play a protective role in static mental health status, fathers’ emotional warmth may function as a unique catalyst for recovery and positive mental developmental change. Actually, studies already found that parenting styles are associated with adolescent overall mental problems [64]. Previous studies also indicated that compared with adolescents with positive maternal parenting styles, adolescents with positive paternal parenting styles have lower scores on overall mental problems [65]. Besides, although prior research largely supports the positive role of parental warmth on adolescent mental health, emerging evidence suggests that paternal warmth may exert unique and sometimes stronger effects in certain relational contexts [66]. For instance, in a sample of juvenile female offenders, paternal warmth exhibited greater explanatory power than maternal warmth in relation to daughters’ psychological outcomes [57]. This aligns qualitatively with our findings, in which only fathers’ emotional warmth, but not mothers’, was linked to more favorable adolescent mental problem profiles.

In addition, we also found that mothers’ over-protection and rejection, rather than fathers’ negative parenting, was more likely to predict adolescents’ translation to the moderate or severe mental problem profiles. This was also found in a network analysis that maternal hostility and harshness were core predictors that dynamically interacted with adolescent mental problems [67]. Complementary research has linked mother-reported overprotection with heightened internalizing and externalizing symptoms and with frustration of adolescents’ psychological needs such as autonomy, relatedness, and competence [68]. Our research found that mothers’ rejection was more harmful to adolescents’ mental health than over-protection. Previous studies also showed that mother’s rejection was more strongly associated with adolescent mental problems like internet gaming disorder compared to over-protection [69]. However, other studies have indicated that maternal over-protection is more strongly associated with personality disorders (another type of mental problem) [70]. These differences may be due to different types of psychological problems. Future research should explore a wider range of mental health issues to more comprehensively understand the associations between various parenting styles and adolescent mental problems.

However, most of the above existing studies belong to the variable-centered approach, focusing on the statistical relationship between variables. They are suitable for overall trend and relationship analysis, but usually ignore the heterogeneity between different individuals or subgroups [71]. By identifying and tracking cross-temporal transitions among latent profiles, the LTA method can simultaneously capture heterogeneous population structures and developmental dynamics [72]. This makes it particularly well suited to addressing the high comorbidity of adolescent mental problems and to providing a more comprehensive understanding of the relationship between parental parenting styles and adolescent mental health [73]. This may explain the discrepancies found between our multilevel model and the LTA model. Future research should perhaps consider applying this person-centered LTA method more.

### Strengths and limitations

This study has several notable strengths. The large sample size, comprehensive assessment of adolescent mental problems, and longitudinal cohort design with two time points enhance the reliability and validity of our findings. To our knowledge, this may be the first study to explore the overall trajectories of adolescent mental problems using ten distinct indicators. Furthermore, our research is among the few that examines the separate association between fathers’ and mothers’ parenting styles and adolescent mental health.

Several limitations must be considered. First, the use of subjective scales for assessing mental problems may lead to potential misclassification in diagnosing adolescent conditions. However, structured questionnaires have practical advantages in large-scale studies. Furthermore, the scales used in this study have demonstrated sufficient reliability and validity (Cronbach’s α = 0.72–0.95), effectively reflecting mental problems among Chinese adolescents. Second, our study focused solely on adolescents in Shenzhen, and the sample is not representative when extrapolating the results. Future research should consider including adolescents from more regions.

## Conclusion

This study reaffirms the protective role of positive parenting styles in adolescents’ mental well-being and the detrimental impact of negative parenting styles. We also described adolescent’s overall mental problem trajectories based on ten mental problems which significantly influenced by paternal emotional warmth, and maternal rejection and over-protection. Our study highlights that, contrary to the traditional Chinese view that men handle external affairs while women manage the home, thereby downplaying fathers’ parenting, paternal involvement is equally vital in adolescents’ mental problem transitions. Public health interventions should emphasize the need for balanced, supportive parenting, particularly in adolescence, to prevent severe mental health issues. Furthermore, the study’s results call for future research to adopt a comprehensive perspective on adolescent mental problems, taking into account various parenting styles and their long-term effects.

## Supplementary Information

Supplementary Material 1.

## Data Availability

The datasets used and/or analysed during the current study are available from the corresponding author on reasonable request.

## References

[1] Caspi A, Houts RM, Ambler A et al. Longitudinal Assessment of Mental Health Disorders and Comorbidities Across 4 Decades Among Participants in the Dunedin Birth Cohort Study. Jama Netw Open 2020; 3(4). 10.1001/jamanetworkopen.2020.3221 PMC7175086 32315069

[2] Solmi M, Radua J, Olivola M, et al. Age at onset of mental disorders worldwide: large-scale meta-analysis of 192 epidemiological studies. Mol Psychiatr. 2022;27(1):281–95. 10.1038/s41380-021-01161-7 PMC8960395 34079068

[3] Morneau-Vaillancourt G, Palaiologou E, Polderman TJC, Eley TC. Research Review: A review of the past decade of family and genomic studies on adolescent mental health. J Child Psychol Psychiatry. 2025;66(6):910–27. 39697100 10.1111/jcpp.14099 PMC12062863

[4] Merikangas KR, He JP, Burstein M, et al. Lifetime prevalence of mental disorders in U.S. adolescents: results from the National Comorbidity Survey Replication–Adolescent Supplement (NCS-A). J Am Acad Child Adolesc Psychiatry. 2010;49(10):980–9. 20855043 10.1016/j.jaac.2010.05.017 PMC2946114

[5] Kieling C, Baker-Henningham H, Belfer M, et al. Child and adolescent mental health worldwide: evidence for action. Lancet. 2011;378(9801):1515–25. 22008427 10.1016/S0140-6736(11)60827-1

[6] Whiteford HA, Degenhardt L, Rehm J, et al. Global burden of disease attributable to mental and substance use disorders: findings from the Global Burden of Disease Study 2010. Lancet. 2013;382(9904):1575–86. 23993280 10.1016/S0140-6736(13)61611-6

[7] Polderman TJ, Benyamin B, de Leeuw CA, et al. Meta-analysis of the heritability of human traits based on fifty years of twin studies. Nat Genet. 2015;47(7):702–9. 25985137 10.1038/ng.3285

[8] Guloksuz S, van Os J, Rutten BPF. The Exposome Paradigm and the Complexities of Environmental Research in Psychiatry. JAMA Psychiatry. 2018;75(10):985–6. 29874362 10.1001/jamapsychiatry.2018.1211

[9] Kallay E, Cheie L. Can I still blame my parents? Links between perceived parenting, cognitive emotion regulation strategies, and adolescent mental health. Curr Psychol. 2023;42(31):27259–74.

[10] Morris AS, Silk JS, Steinberg L, Myers SS, Robinson LR. The Role of the Family Context in the Development of Emotion Regulation. Soc Dev. 2007;16(2):361–88. 19756175 10.1111/j.1467-9507.2007.00389.x PMC2743505

[11] Rothenberg WA, Lansford JE, Bornstein MH, et al. Effects of Parental Warmth and Behavioral Control on Adolescent Externalizing and Internalizing Trajectories Across Cultures. J Res Adolesc. 2020;30(4):835–55. 32609411 10.1111/jora.12566 PMC8059478

[12] Manuele SJ, Yap MBH, Lin S, Pozzi E, Whittle S. Associations between paternal versus maternal parenting behaviors and child and adolescent internalizing problems: A systematic review and meta-analysis. Clin Psychol Rev 2023; 105. 10.1016/j.cpr.2023.102339 37793269

[13] Arslan IB, Lucassen N, Keijsers L, Stevens G. When Too Much Help is of No Help: Mothers’ and Fathers’ Perceived Overprotective Behavior and (Mal)Adaptive Functioning in Adolescents. J Youth Adolesc. 2023;52(5):1010–23. 36633796 10.1007/s10964-022-01723-0 PMC10027782

[14] Tavassolie T, Dudding S, Madigan AL, Thorvardarson E, Winsler A. Differences in Perceived Parenting Style Between Mothers and Fathers: Implications for Child Outcomes and Marital Conflict. J Child Fam stud. 2016;25(6):2055–68.

[15] Lee SJ, Pace GT, Lee JY, Knauer H. The Association of Fathers’ Parental Warmth and Parenting Stress to Child Behavior Problems. Child Youth Serv Rev. 2018;91:1–10. 31662592 10.1016/j.childyouth.2018.05.020 PMC6818750

[16] Andreas A, White LO, Sierau S, Perren S, von Klitzing K, Klein AM. Like mother like daughter, like father like son? Intergenerational transmission of internalizing symptoms at early school age: a longitudinal study. Eur Child Adolesc Psychiatry. 2018;27(8):985–95. 29302748 10.1007/s00787-017-1103-y

[17] Field AP, Lester KJ, Cartwright-Hatton S et al. Maternal and paternal influences on childhood anxiety symptoms: A genetically sensitive comparison. J Appl Dev Psychol 2020; 68. 10.1016/j.appdev.2020.101123 PMC7377633 32704198

[18] Yaffe Y. Systematic review of the differences between mothers and fathers in parenting styles and practices. Curr Psychol. 2023;42(19):16011–24.

[19] Kessler RC, Avenevoli S, McLaughlin KA, et al. Lifetime co-morbidity of DSM-IV disorders in the US National Comorbidity Survey Replication Adolescent Supplement (NCS-A). Psychol Med. 2012;42(9):1997–2010. 22273480 10.1017/S0033291712000025 PMC3448706

[20] Axelson DA, Birmaher B. Relation between anxiety and depressive disorders in childhood and adolescence. Depress Anxiety. 2001;14(2):67–78. 11668659 10.1002/da.1048

[21] Kessler RC, Amminger GP, Aguilar-Gaxiola S, Alonso J, Lee S, Ustun TB. Age of onset of mental disorders: a review of recent literature. Curr Opin Psychiatry. 2007;20(4):359–64. 17551351 10.1097/YCO.0b013e32816ebc8c PMC1925038

[22] Rapee RM, Oar EL, Johnco CJ, et al. Adolescent development and risk for the onset of social-emotional disorders: A review and conceptual model. Behav Res Ther. 2019;123:103501. 31733812 10.1016/j.brat.2019.103501

[23] Krueger RF, Eaton NR. Transdiagnostic factors of mental disorders. World Psychiatry. 2015;14(1):27–9. 25655146 10.1002/wps.20175 PMC4329885

[24] Pinquart M. Associations of Parenting Dimensions and Styles with Internalizing Symptoms in Children and Adolescents: A Meta-Analysis. Marriage Family Rev. 2017;53(7):613–40.

[25] Copeland WE, Wolke D, Shanahan L, Costello EJ. Adult Functional Outcomes of Common Childhood Psychiatric Problems: A Prospective, Longitudinal Study. JAMA Psychiatry. 2015;72(9):892–9. 26176785 10.1001/jamapsychiatry.2015.0730 PMC4706225

[26] Gobel K, Ortelbach N, Cohrdes C, et al. Co-occurrence, stability and manifestation of child and adolescent mental health problems: a latent transition analysis. BMC Psychol. 2022;10(1):267. 36376939 10.1186/s40359-022-00969-4 PMC9664619

[27] Kennedy SM, Tonarely NA, Halliday E, Ehrenreich-May J. A person-centered approach to understanding heterogeneity of youth receiving transdiagnostic treatment for emotional disorders. J Consult Clin Psychol. 2022;90(3):234–45. 35175069 10.1037/ccp0000710

[28] Bergman LR, Magnusson D. A person-oriented approach in research on developmental psychopathology. Dev Psychopathol. 1997;9(2):291–319. 9201446 10.1017/s095457949700206x

[29] von Eye A, Bergman LR. Research strategies in developmental psychopathology: dimensional identity and the person-oriented approach. Dev Psychopathol. 2003;15(3):553–80. 14582932 10.1017/s0954579403000294

[30] Teuber Z, Tang X, Sielemann L, Otterpohl N, Wild E. Autonomy-related Parenting Profiles and their Effects on Adolescents’ Academic and Psychological Development: A Longitudinal Person-oriented Analysis. J Youth Adolesc. 2022;51(7):1333–53. 34807340 10.1007/s10964-021-01538-5 PMC9135772

[31] Smetana JG. Current research on parenting styles, dimensions, and beliefs. Curr Opin Psychol. 2017;15:19–25. 28813261 10.1016/j.copsyc.2017.02.012

[32] Kassis W, Vasiou A, Aksoy D, Favre CA, Talmon-Gros Artz S, Magnusson D. Parenting style patterns and their longitudinal impact on mental health in abused and nonabused adolescents. Front Psychiatry. 2025;16:1548549. 40099147 10.3389/fpsyt.2025.1548549 PMC11911485

[33] Colizzi M, Lasalvia A, Ruggeri M. Prevention and early intervention in youth mental health: is it time for a multidisciplinary and trans-diagnostic model for care? Int J Ment Health Syst. 2020;14:23. 32226481 10.1186/s13033-020-00356-9 PMC7092613

[34] Wang P, Wang Z, Qiu S. Universal, school-based transdiagnostic interventions to promote mental health and emotional wellbeing: a systematic review. Child Adolesc Psychiatry Ment Health. 2024;18(1):47. 38600562 10.1186/s13034-024-00735-x PMC11007989

[35] Wang S, Yu M, Zhang X, et al. Comparison of mental health networks across different educational stages. Eur Child Adolesc Psychiatry. 2025;34(10):3115–23. 40314827 10.1007/s00787-025-02731-8

[36] Wang J, Li Y, He E. Development and standardization of mental health scale for middle school students in China. Psychosoc Sci. 1997;4:15–20.

[37] Kroenke K, Spitzer RL, Williams JB. The PHQ-9: validity of a brief depression severity measure. J Gen Intern Med. 2001;16(9):606–13. 11556941 10.1046/j.1525-1497.2001.016009606.x PMC1495268

[38] Sun J, Liang K, Chi X, Chen S. Psychometric Properties of the Generalized Anxiety Disorder Scale-7 Item (GAD-7) in a Large Sample of Chinese Adolescents. Healthc (Basel) 2021; 9(12). 10.3390/healthcare9121709 PMC8701121 34946435

[39] Spitzer RL, Kroenke K, Williams JB, Lowe B. A brief measure for assessing generalized anxiety disorder: the GAD-7. Arch Intern Med. 2006;166(10):1092–7. 16717171 10.1001/archinte.166.10.1092

[40] Wang W, Bian Q, Zhao Y, et al. Reliability and validity of the Chinese version of the Patient Health Questionnaire (PHQ-9) in the general population. Gen Hosp Psychiatry. 2014;36(5):539–44. 25023953 10.1016/j.genhosppsych.2014.05.021

[41] Jiang J, Lu Z, Jiang B-J, Xu Y. Revision of the short-form Egna Minnen av Barndoms Uppfostran for Chinese. Psychol Dev Educ. 2010;26(1):94–9.

[42] Johnson SK. Latent profile transition analyses and growth mixture models: A very non-technical guide for researchers in child and adolescent development. New Dir Child Adoles. 2021;175:111–39. 10.1002/cad.20398 33634554

[43] Berlin KS, Parra GR, Williams NA. An Introduction to Latent Variable Mixture Modeling (Part 2): Longitudinal Latent Class Growth Analysis and Growth Mixture Models. J Pediatr Psychol. 2014;39(2):188–203. 24277770 10.1093/jpepsy/jst085

[44] Sinha P, Calfee CS, Delucchi KL. Practitioner’s Guide to Latent Class Analysis: Methodological Considerations and Common Pitfalls. Crit Care Med. 2021;49(1):e63–79. 33165028 10.1097/CCM.0000000000004710 PMC7746621

[45] Vidotto D, Vermunt JK, Van Deun K. Multiple imputation of longitudinal categorical data through bayesian mixture latent Markov models. J Appl Stat. 2020;47(10):1720–38. 35707130 10.1080/02664763.2019.1692794 PMC9041790

[46] Satorra A, Bentler PM. Ensuring Positiveness of the Scaled Difference Chi-square Test Statistic. Psychometrika. 2010;75(2):243–8. 20640194 10.1007/s11336-009-9135-y PMC2905175

[47] Ren Y, Zhang S, Huang C, Zhang J, Jiang T, Fang Y. Perceived parental rearing styles and depression in Chinese adolescents: the mediating role of self-compassion. Front Psychiatry. 2024;15:1417355. 39364381 10.3389/fpsyt.2024.1417355 PMC11446764

[48] Wolfradt U, Hempel S, Miles JNV. Perceived parenting styles, depersonalisation, anxiety and coping behaviour in adolescents. Pers Indiv Differ. 2003;34(3):521–32.

[49] Galvan A. Adolescence, brain maturation and mental health. Nat Neurosci. 2017;20(4):503–4. 28352110 10.1038/nn.4530

[50] LeDoux JE, Pine DS. Using Neuroscience to Help Understand Fear and Anxiety: A Two-System Framework. Am J Psychiatry. 2016;173(11):1083–93. 27609244 10.1176/appi.ajp.2016.16030353

[51] Romund L, Raufelder D, Flemming E, et al. Maternal parenting behavior and emotion processing in adolescents-An fMRI study. Biol Psychol. 2016;120:120–5. 27645501 10.1016/j.biopsycho.2016.09.003

[52] Guyer AE, Jarcho JM, Perez-Edgar K, et al. Temperament and Parenting Styles in Early Childhood Differentially Influence Neural Response to Peer Evaluation in Adolescence. J Abnorm Child Psychol. 2015;43(5):863–74. 25588884 10.1007/s10802-015-9973-2 PMC4468038

[53] Chu XW, Chen ZK. The Associations Between Parenting and Bullying Among Children and Adolescents: A Systematic Review and Meta-Analysis. J Youth Adolescence 2024. 10.1007/s10964-024-02108-1 39549118

[54] Verhoeven M, Bogels SM, van der Bruggen CC. Unique Roles of Mothering and Fathering in Child Anxiety; Moderation by Child’s Age and Gender. J Child Fam Stud. 2012;21(2):331–43. 22448108 10.1007/s10826-011-9483-y PMC3304056

[55] Majdandzic M, de Vente W, Colonnesi C, Bogels SM. Fathers’ challenging parenting behavior predicts less subsequent anxiety symptoms in early childhood. Behav Res Ther. 2018;109:18–28. 30077804 10.1016/j.brat.2018.07.007

[56] Zhou H, Tian CY, Hong LW, Fan ZG, Chen W. Relationship between Parenting Style and Peer Relationships during Early Adolescence: The Mediating Role of Parental Mentalizing. J Genet Psychol 2024. 10.1080/00221325.2024.2413488 39400539

[57] Moller EL, Nikolic M, Majdandzic M, Bogels SM. Associations between maternal and paternal parenting behaviors, anxiety and its precursors in early childhood: A meta-analysis. Clin Psychol Rev. 2016;45:17–33. 26978324 10.1016/j.cpr.2016.03.002

[58] Lee SK, Gewirtz AH, Piehler TF. Parenting Profiles in Military Families: Intervention-Related Transitions and Relationships to Child Adjustment. Prev Sci. 2024;25(7):1040–52. 39285084 10.1007/s11121-024-01721-7 PMC11519299

[59] Putnick DL, Bornstein MH, Lansford JE, et al. Agreement in Mother and Father Acceptance-Rejection, Warmth, and Hostility/Rejection/Neglect of Children across Nine Countries. Cross Cult Res. 2012;46(3):191–223. 23024576 10.1177/1069397112440931 PMC3457062

[60] Pan B, Wang Y, Xu P, et al. The complex longitudinal influence of paternal and maternal parental psychological flexibility on child problem behavior: exploring the role of parenting styles. BMC Psychol. 2024;12(1):793. 39736784 10.1186/s40359-024-02291-7 PMC11684130

[61] Dai YL, Zheng Y, Hu KS, et al. Heterogeneity in the co-occurrence of depression and anxiety among adolescents: Results of latent profile analysis. J Affect Disorders. 2024;357:77–84. 38670464 10.1016/j.jad.2024.04.065

[62] Xie C, Xiang S, Shen C, et al. A shared neural basis underlying psychiatric comorbidity. Nat Med. 2023;29(5):1232–42. 37095248 10.1038/s41591-023-02317-4 PMC10202801

[63] Wight RG, SepÚlveda JE, Aneshensel CS. Depressive symptoms: how do adolescents compare with adults? J Adolesc Health. 2004;34(4):314–23. 15041001 10.1016/j.jadohealth.2003.05.003

[64] Yap MB, Jorm AF. Parental factors associated with childhood anxiety, depression, and internalizing problems: a systematic review and meta-analysis. J Affect Disord. 2015;175:424–40. 25679197 10.1016/j.jad.2015.01.050

[65] Azman Ö, Mauz E, Reitzle M, Geene R, Hölling H, Rattay P. Associations between Parenting Style and Mental Health in Children and Adolescents Aged 11–17 Years: Results of the KiGGS Cohort Study (Second Follow-Up). Children-Basel 2021; 8(8). 10.3390/children8080672 PMC8394813 34438563

[66] Peng B, Hu NN, Yu HY, Xiao HS, Luo J. Parenting Style and Adolescent Mental Health: The Chain Mediating Effects of Self-Esteem and Psychological Inflexibility. Front Psychol 2021; 12. 10.3389/fpsyg.2021.738170 PMC8548717 34721210

[67] Sun X, Yuan T, Chen F, Li Y, Jiang N. Network analysis of maternal parenting practices and adolescent mental health problems: a longitudinal study. Child Adolesc Psychiatry Ment Health. 2024;18(1):38. 38504321 10.1186/s13034-024-00728-w PMC10953267

[68] Van Petegem S, Antonietti JP, Nunes CE, Kins E, Soenens B. The Relationship between Maternal Overprotection, Adolescent Internalizing and Externalizing Problems, and Psychological Need Frustration: A Multi-Informant Study Using Response Surface Analysis. J Youth Adolescence. 2020;49(1):162–77. 10.1007/s10964-019-01126-8 31583507

[69] Zhang LN, Han JQ, Liu MQ, Yang C, Liao YH. The prevalence and possible risk factors of gaming disorder among adolescents in China. BMC Psychiatry 2024; 24(1). 10.1186/s12888-024-05826-9 PMC11110186 38773555

[70] Cheng HG, Huang Y, Liu Z, Liu B. Associations linking parenting styles and offspring personality disorder are moderated by parental personality disorder, evidence from China. Psychiatry Res. 2011;189(1):105–9. 21195487 10.1016/j.psychres.2010.12.006

[71] Daljeet KN, Bremner NL, Giammarco EA, Meyer JP, Paunonen SV. Taking a person-centered approach to personality: A latent-profile analysis of the HEXACO model of personality. J Res Pers. 2017;70:241–51.

[72] Abarda A, Dakkon M, Azhari M, Zaaloul A, Khabouze M. Latent Transition Analysis (LTA): A Method for Identifying Differences in Longitudinal Change Among Unobserved Groups. Procedia Comput Sci. 2020;170:1116–21.

[73] Magson NR, van Zalk N, Mörtberg E, Chard I, Tillfors M, Rapee RM. Latent stability and change in subgroups of social anxiety and depressive symptoms in adolescence: A latent profile and transitional analysis. J Anxiety Disord 2022; 87. 10.1016/j.janxdis.2022.102537 35168001

