# Supplementary material for: The association between maternal and paternal parenting styles and adolescents’ overall mental problem: a longitudinal study
Source: BMC Psychol. 2026 May 19;14:1035. doi: 10.1186/s40359-026-04525-2 (PMC13352710; doi:10.1186/s40359-026-04525-2)
Supplement: Supplementary file 1 — Supplementary Material 1. [file 40359_2026_4525_MOESM1_ESM.docx]

etable1. Results of assessment of adolescent mental problems

| Psychological problems | No problems  N (%) | Mild problems  N (%) | Moderate problems  N (%) | Severe problems  N (%) | Serious problems  N (%) |
| --- | --- | --- | --- | --- | --- |
| MMHI-60 | | |  |  |  |
| Obsessive-Compulsive tendencies | | |  |  |  |
| Baseline | 5068 (57.32%) | 2986 (33.77%) | 715 (8.09%) | 66 (0.75%) | 7 (0.08%) |
| One-year follow-up | 4981 (56.33%) | 2962 (33.50%) | 830 (9.39%) | 64 (0.72%) | 5 (0.06%) |
| Paranoid Ideation | |  |  |  |  |
| Baseline | 6350 (71.82%) | 1891 (21.39%) | 531 (6.01%) | 62 (0.70%) | 8 (0.09%) |
| One-year follow-up | 6013 (68.00%) | 2105 (23.81%) | 634 (7.17%) | 79 (0.89%) | 11 (0.12%) |
| Hostility |  |  |  |  |  |
| Baseline | 6469 (73.16%) | 1631 (18.45%) | 597 (6.75%) | 122 (1.38%) | 23 (0.26%) |
| One-year follow-up | 6207 (70.20%) | 1799 (20.35%) | 665 (7.52%) | 150 (1.70%) | 21 (0.24%) |
| Interpersonal Sensitivity | |  |  |  |  |
| Baseline | 5764 (65.19%) | 2307 (26.09%) | 692 (7.83%) | 72 (0.81%) | 7 (0.08%) |
| One-year follow-up | 5475 (61.92%) | 2478 (28.03%) | 800 (9.05%) | 84 (0.95%) | 5 (0.06%) |
| Academic Stress | |  |  |  |  |
| Baseline | 4672 (52.84%) | 2662 (30.11%) | 1130 (12.78%) | 334 (3.78%) | 44 (0.50%) |
| One-year follow-up | 4255 (48.12%) | 2768 (31.31%) | 1382 (15.63%) | 387 (4.38%) | 50 (0.57%) |
| Maladaptation |  |  |  |  |  |
| Baseline | 6222 (70.37%) | 2046 (23.14%) | 524 (5.93%) | 47 (0.53%) | 3 (0.03%) |
| One-year follow-up | 5632 (63.70%) | 2494 (28.21%) | 644 (7.28%) | 67 (0.76%) | 5 (0.06%) |
| Emotional Disturbance | |  |  |  |  |
| Baseline | 5107 (57.76%) | 2744 (31.03%) | 863 (9.76%) | 117 (1.32%) | 11 (0.12%) |
| One-year follow-up | 4672 (52.84%) | 2938 (33.23%) | 1079 (12.20%) | 141 (1.59%) | 12 (0.14%) |
| Psychological Imbalance | |  |  |  |  |
| Baseline | 7228 (81.75%) | 1300 (14.70%) | 292 (3.30%) | 16 (0.18%) | 6 (0.07%) |
| One-year follow-up | 6975 (78.88%) | 1513 (17.11%) | 332 (3.75%) | 17 (0.19%) | 5 (0.06%) |
| GAD-7 |  |  |  |  |  |
| Anxiety | No anxiety | Mild anxiety | Moderate anxiety | Severe anxiety |  |
| Baseline | 5521 (62.44%) | 2251 (25.46%) | 689 (7.79%) | 381 (4.31%) |  |
| One-year follow-up | 5116 (57.86%) | 2587 (29.26%) | 788 (8.91%) | 351 (3.97%) |  |
| PHQ-9 |  |  |  |  |  |
| Depression | No depression | Mild depression | Moderate depression | Moderately severe depression | Severe depression |
| Baseline | 4934 (55.80%) | 2492 (28.18%) | 776 (8.78%) | 411 (4.65%) | 229 (2.59%) |
| One-year follow-up | 4395 (49.71%) | 2855 (32.29%) | 863 (9.76%) | 508 (5.75%) | 221 (2.50%) |

Abbreviation: MMHI-60, Mental Health Inventory of Middle School Students; GAD-7, the Generalized Anxiety Disorder 7-item Scale, PHQ-9, Patient Health Questionnaire Depression Scale

etable2. Differences in demographic variables and parenting styles scores between fathers and mothers

| Variable | Father | Mother | *P-value* |
| --- | --- | --- | --- |
| Education level, No. (%) | |  | <0.01 |
| ≤high school | 6018 (68.1%) | 6116 (69.2%) |  |
| > high school | 2824 (31.9%) | 2726 (30.8%) |  |
| Baseline score of parenting style, mean (SD) | | |  |
| Rejection | 1.53 (0.58) | 1.54 (0.56) | 0.28 |
| Emotional warmth | 2.48 (0.76) | 2.59 (0.75) | <0.01 |
| Over-protection | 1.98 (0.52) | 2.07 (0.54) | <0.01 |
| One year follow-up score of parenting style, mean (SD) | | |  |
| Rejection | 1.49 (0.55) | 1.49 (0.52) | 0.52 |
| Emotional warmth | 2.48 (0.76) | 2.60 (0.74) | <0.01 |
| Over-protection | 1.99 (0.51) | 2.07 (0.53) | <0.01 |

Abbreviation: Standard deviation

etable3. Correlation analysis between maternal and paternal parenting scores

|  | Father's rejection | Father's emotional warmth | Father's over-protection | Mother's rejection | Mother's emotional warmth | Mother's over-protection |
| --- | --- | --- | --- | --- | --- | --- |
| Father's rejection | 1.00 | -0.35 | 0.61 | 0.76 | -0.25 | 0.50 |
| Father's emotional warmth | -0.35 | 1.00 | -0.11 | -0.29 | 0.86 | -0.10 |
| Father's over-protection | 0.61 | -0.11 | 1.00 | 0.52 | -0.09 | 0.84 |
| Mother's rejection | 0.76 | -0.29 | 0.52 | 1.00 | -0.35 | 0.61 |
| Mother's emotional warmth | -0.25 | 0.86 | -0.09 | -0.35 | 1.00 | -0.11 |
| Mother's over-protection | 0.50 | -0.10 | 0.84 | 0.61 | -0.11 | 1.00 |

eTable4. Fit indices for models testing measurement invariance between baseline and follow-up

|  | Free parameters | AIC | BIC | Loglikelihood | df | *P* |
| --- | --- | --- | --- | --- | --- | --- |
| Measurement invariant | 37 | 230257 | 231143 | -115003 |  |  |
| Non-measurement invariant | 58 | 230180 | 231895 | -114848 | 21 | 0.99 |

Abbreviation: AIC, Akaike Information Criterion; BIC, Bayesian Information Criterion. Values in bold indicate the best-fitting model.

eTable5 Cross-sectional associations between parenting style scores and mental problem profiles ^a b^

| Parenting style [OR (95%CI)] | Latent status | | |
| --- | --- | --- | --- |
|  | Low mental problem | Moderate mental problem | Severe mental problem |
| Baseline |  |  |  |
| Father’s rejection | Ref. | **2.30 (2.20, 2.44)** | **3.74 (3.48, 4.02)** |
| Father’s emotional warmth | Ref. | **0.68 (0.65, 0.71)** | **0.50 (0.47, 0.54)** |
| Father’s over-protection | Ref. | **1.89 (1.79, 2.00)** | **3.02 (2.81, 3.25)** |
| Mother’s rejection | Ref. | **2.31 (2.19, 2.44)** | **3.88 (3.61, 4.18)** |
| Mother’s emotional warmth | Ref. | **0.71 (0.67, 0.74)** | **0.52 (0.48, 0.55)** |
| Mother’s over-protection | Ref. | **1.99 (1.88, 2.10)** | **3.18 (2.95, 3.43)** |
| One year follow-up |  |  |  |
| Father’s rejection | Ref. | **1.61 (1.53, 1.69)** | **2.22 (2.07, 2.38)** |
| Father’s emotional warmth | Ref. | **0.75 (0.71, 0.79)** | **0.57 (0.53, 0.61)** |
| Father’s over-protection | Ref. | **1.47 (1.40, 1.55)** | **1.97 (1.84, 2.11)** |
| Mother’s rejection | Ref. | **1.63 (1.55, 1.72)** | **2.24 (2.09, 2.40)** |
| Mother’s emotional warmth | Ref. | **0.76 (0.72, 0.80)** | **0.58 (0.55, 0.62)** |
| Mother’s over-protection | Ref. | **1.49 (1.42, 1.57)** | **2.02 (1.88, 2.16)** |

Abbreviation: CI, Confidence interval

^a^ Model was adjusted for child age, sex, school, preterm birth or not, parental education levels, only child, single-parent families.

^b^ Bold indicates that the *P value* < 0.05

eTable6. *t* Tests examining whether the change in parenting style scores between baseline and follow-up for each transition profile significantly differed from zero ^a^

| Change in parenting style scores | Transition pattern [mean (SD)] | | |
| --- | --- | --- | --- |
|  | L→L | L→M | L→S |
| Father’s rejection | **-0.029 (0.421)** | **0.179 (0.524)** | **0.374 (0.629)** |
| Mother’s rejection | **-0.031 (0.425)** | **0.181 (0.504)** | **0.371 (0.686)** |
| Father’s over-protection | 0.007 (0.498) | **0.166 (0.504)** | **0.430 (0.606)** |
| Mother’s over-protection | 0.013 (0.516) | **0.199 (0.523)** | **0.396 (0.615)** |
| Father’s emotional warmth | 0.006 (0.786) | **-0.128 (0.698)** | **-0.333 (0.834)** |
| Mother’s emotional warmth | 0.006 (0.782) | **-0.094 (0.700)** | **-0.312 (0.813)** |
|  | M→M | M→L | M→S |
| Father’s rejection | **-0.308 (0.583)** | **-0.070 (0.516)** | **0.141 (0.672)** |
| Mother’s rejection | **-0.314 (0.539)** | **-0.067 (0.476)** | **0.135 (0.600)** |
| Father’s over-protection | **-0.160 (0.541)** | -0.023 (0.480) | **0.132 (0.568)** |
| Mother’s over-protection | **-0.188 (0.522)** | **-0.031 (0.474)** | **0.126 (0.555)** |
| Father’s emotional warmth | **0.188 (0.816)** | 0.011 (0.609) | **-0.057 (0.652)** |
| Mother’s emotional warmth | **0.138 (0.796)** | 0.026 (0.585) | -0.050 (0.640) |
|  | S→S | S→L | S→M |
| Father’s rejection | **-0.653 (0.837)** | **-0.310 (0.681)** | **-0.116 (0.711)** |
| Mother’s rejection | **-0.658 (0.810)** | **-0.356 (0.682)** | **-0.130 (0.698)** |
| Father’s over-protection | **-0.409 (0.749)** | **-0.201 (0.575)** | **-0.059 (0.621)** |
| Mother’s over-protection | **-0.471 (0.716)** | **-0.216 (0.558)** | **-0.057 (0.597)** |
| Father’s emotional warmth | 0.044 (0.906) | **0.116 (0.698)** | 0.041 (0.624) |
| Mother’s emotional warmth | -0.011 (0.910) | **0.152 (0.711)** | **0.058 (0.660)** |

Abbreviation: CI, Confidence interval; L, low mental problem Profile; M, moderate mental problem profile; S, severe mental problem profile

^a^ Values in bold indicated the change score was significantly different from zero (*P value* < 0.05).

eTable7 Associations between baseline parenting style scores and one year follow-up mental problem profiles ^a b^

| Parenting style [OR (95%CI)] | Latent status | | |
| --- | --- | --- | --- |
|  | Low mental problem | Moderate mental problem | Severe mental problem |
| Father’s rejection | Ref. | **2.45 (2.23, 2.69)** | **4.37 (3.87, 4.92)** |
| Father’s emotional warmth | Ref. | **0.64 (0.59, 0.71)** | **0.50 (0.44, 0.56)** |
| Father’s over-protection | Ref. | **2.30 (2.09, 2.53)** | **4.16 (3.69, 4.69)** |
| Mother’s rejection | Ref. | **2.56 (2.33, 2.82)** | **5.00 (4.43, 5.64)** |
| Mother’s emotional warmth | Ref. | **0.67 (0.61, 0.73)** | **0.50 (0.44, 0.56)** |
| Mother’s over-protection | Ref. | **2.37 (2.15, 2.62)** | **4.41 (3.90, 4.98)** |

Abbreviation: CI, Confidence interval

^a^ Model was adjusted for child age, sex, school, preterm birth or not, parental education levels, only child, single-parent families.

^b^ Bold indicates that the *P value* < 0.05

Analytical Procedure and Code for Measurement Invariance:

Measurement invariance was tested in R using the slca package to estimate the latent transition models and the SBSDiff package to compute the Satorra-Bentler scaled χ^2^ difference test.

Model 1 (Constrained Model): We estimated the 3-class LTA model where the item response parameters for the ten mental health outcomes (from the MMHI, GAD-7, and PHQ-9) were constrained to be equal across Time 1 (LC1) and Time 2 (LC2). This is achieved using the constraints = c ("LC1", "LC2") argument.

Model 2 (Unconstrained Model): We estimated the identical structural model, but allowed the item response parameters to be freely estimated at each time point by removing the constraint argument.

Model Comparison: We extracted the χ^2^ values, degrees of freedom, and scaling correction factors from both model summaries and utilized the sbs.chi() function to

R Code Implementation:

library(slca)

library(SBSDiff)

# Model 1: Constrained Model (Measurement Invariance assumed)

# The item response probabilities for the 10 indicators are constrained to be equal across LC1 (baseline) and LC2 (follow-up).

model1 <- slca(

LC1[3] ~ b_qp_level.x + b_pz_level.x + b_dd_level.x + b_rj_level.x + b_xx_level.x + b_sy_level.x + b_qx_level.x + b_xl_level.x + d_level.x + f_level.x,

LC2[3] ~ b_qp_level.y + b_pz_level.y + b_dd_level.y + b_rj_level.y + b_xx_level.y + b_sy_level.y + b_qx_level.y + b_xl_level.y + d_level.y + f_level.y,

LC1 ~ LC2,

constraints = c("LC1", "LC2")

) %>% estimate(data = data_gm_twoyears2)

# Model 2: Unconstrained Model (Freely estimated)

# The constraints argument is removed, allowing parameters to vary across time points.

model2 <- slca(

LC1[3] ~ b_qp_level.x + b_pz_level.x + b_dd_level.x + b_rj_level.x + b_xx_level.x + b_sy_level.x + b_qx_level.x + b_xl_level.x + d_level.x + f_level.x,

LC2[3] ~ b_qp_level.y + b_pz_level.y + b_dd_level.y + b_rj_level.y + b_xx_level.y + b_sy_level.y + b_qx_level.y + b_xl_level.y + d_level.y + f_level.y,

LC1 ~ LC2

) %>% estimate(data = data_gm_twoyears2)

# Extract summaries

summary(model1)

summary(model2)

# Calculate Satorra-Bentler scaled chi-square difference using parameters from the summaries

# (chi0/chi1 = chi-square values; df0/df1 = degrees of freedom; c0/c1 =`r

#sbs.chi(chi0, chi1, df0, df1, c0, c1)
